# Effect of water-jet flossing on surface roughness and color stability of dental resin-based composites

**DOI:** 10.4317/jced.56153

**Published:** 2020-02-01

**Authors:** Mohammed Alharbi, Ra’fat Farah

**Affiliations:** 1 Dentist, Dentistry General Department, Ministry of Health, Qassim, Kingdom of Saudi Arabia; 2 Assistant Professor, Department of Prosthodontics, College of dentistry, Qassim University, Kingdom of Saudi Arabia

## Abstract

**Background:**

The purpose of this *in vitro* study was to investigate the effects of water-jet flossing on the color stability and surface roughness of five resin-based composites.

**Material and Methods:**

Five commercially available composite resins were studied. Nine disc-shaped specimens (6x2mm) were fabricated from each composite. The specimens were randomly allocated into three groups and three different treatments were performed on each group: storage in water (control group), water-jet flossing using 50 Psi water pressure, and water-jet flossing using 100 Psi water pressure. The water-jet flossing was performed in a standardized manner using a Waterpik® Aquarius® water flosser. Color and roughness were measured at baseline and at the end of 30 minutes of treatment, which is approximately equivalent to 5 years of simulated water-jet flossing for 1 minute once a day. The data were statistically analyzed using two-way analysis of variance (ANOVA) with Bonferroni and Tukey’s post-hoc tests.

**Results:**

No significant color change was found after 5 simulated years of water-jet flossing, irrespective of composite type and water-flossing pressure setting (*p* > 0.05). Furthermore, none of composite specimens showed any significant surface roughness changes except for the two composites with spherical filler specimens in the 100 Psi treatment group. These composites exhibited a significant increase in surface roughness compared with the nano-filled composite (*p* = 0.001 and *p* = 0.006). However, the differences were clinically acceptable (≈0.2 µm).

**Conclusions:**

In terms of surface roughness and color, water-jet flossing is safe to be used on composite restorations within the settings of this study.

** Key words:**Color difference (∆E), resin composites, surface roughness, water flosser.

## Introduction

An ideal restorative material should mimic the original tooth in its durability and ability to withstand the stresses of a hostile oral environment. Furthermore, this material should preserve its ideal properties, which include maintaining its smooth surface and maintaining a desired color and gloss (1,2). In addition to inherent material properties, there are many extrinsic factors that affect the color changes and surface deterioration of a restorative material: physical/chemical factors (temperature, pH, moisture, ultraviolet (UV) irradiation, absorption and adsorption of colorants, etc.) and mechanical factors (masticatory load, food bolus abrasiveness, finishing and polishing) (3-5). These mechanical factors also include oral hygiene procedures that may come into contact with the surface of the restorative materials such as a toothbrush and abrasive particles in toothpastes. Many previous studies have evaluated surface changes (color stability, gloss and surface roughness) of different restorative materials on the basis of brushing and abrasive dentifrices (6-10).

Recently, water flossers (also known as oral irrigators) have become popular among patients and are being used more frequently as an adjunctive oral hygiene tool. The idea of water-jet cleaning devices is not new; they were introduced at the beginning of the previous century. The first personal commercially available dental water-jet flosser with accepTable water pressure was introduced in the 1960s (11). Previous studies have shown that water flossers are safe; there is no evidence of unfavorable effects on the attachment or junctional epithelium (12). These devices have also demonstrated effectiveness at removing bacteria and reducing the signs of inflammation with a significant reduction in gingivitis (13,14). Recently, some commercial brands of flossers received the American Dental Association (ADA) Seal of Acceptance for their safety and efficacy for removing plaque and for helping to prevent and reduce gingivitis (15).

Water flossers use a pressurized water-jet to clean plaque and remnants (material alba) from the teeth. The water pressure may reach up to 0.6 MPa. Studies have reported safe use of this water pressure on attachment or junctional epithelium. However, some reports have noted bleeding, pain, and interconnective tissue hemorrhage when the water-jet is used on nonattached (e.g., floor of the mouth) or inflamed tissues (16,17). Enamel is known for its high degree of hardness (around 400Kg/mm2) and, in normal conditions, is not affected by abrasion from teeth brushing with high-abrasive dentifrice (18,19). One would expect that water flosser pressure would not affect the surface of enamel. However, composite base restorative materials, which are inferior to dental enamel in hardness (physical and mechanical properties) and are characterized by a heterogeneous composition, may be negatively affected in terms of color, loss of gloss and an increase in surface roughness (i.e., esthetic failure). Furthermore, an increase in restoration surface roughness may lead to an increase in biofilm and accumulation on the restoration, which may lead to problems including patient discomfort, secondary caries, gingival inflammation, and surface staining (20). In additional, a rough surface of restorations increases the coefficient of friction and may increase the rate of wear (21). Unfortunately, no recent studies, to the best of our knowledge, have examined the effect of using water flossers on the color and surface roughness of dental resin composite restorative materials.

The goal of this study was to investigate the effects of water-jet flossing on the color stability and surface roughness of five resin-based composite dental filling materials. The null hypotheses were first that water-jet flossing would not affect the color stability of resin-based composites and second that water-jet flossing would not affect the surface roughness of resin-based composites.

Material and Methods

-Specimen preparation

**Figure 1:**
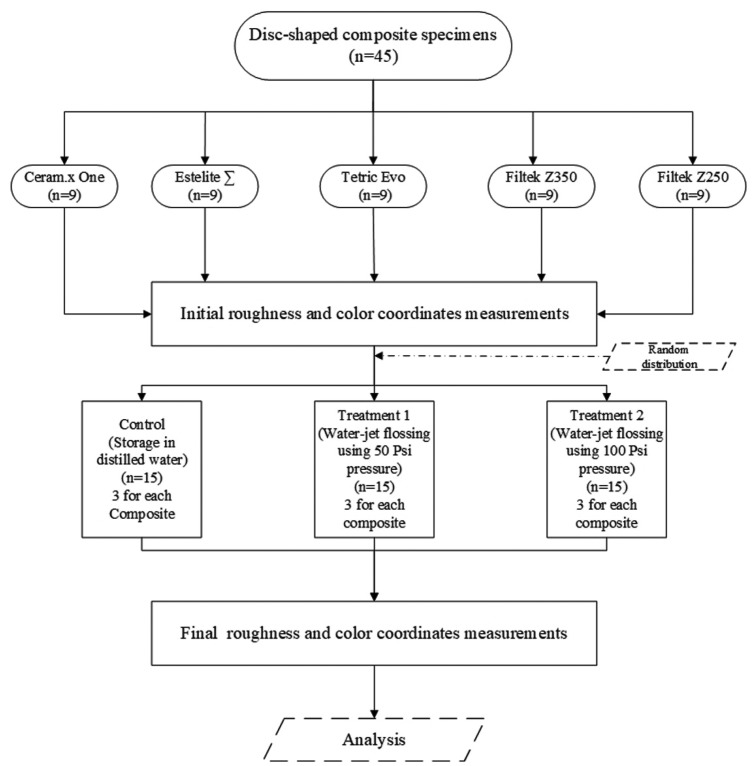
The CONSORT Flow diagram of experimental procedure.

Five commercially available universal resin-based composites with an A2 shade were used in this study, including two contemporary composites with spherical fillers (Ceram.x SphereTEC™ and Estelite® Sigma Quick) one nano-filled composite (Filtek™Z350) and two conventional micro-hybrid composites with irregularly shaped fillers (Filtek™ Z250 and Tetric Evoceram®) ( Table 1). Nine disk-shaped specimens, each measuring 6 mm in diameter and 2 mm in thickness, were prepared for each composite resin (i.e., a total of 45 specimens) (Fig. 1). The number of specimens in each group was determined after performing power analyses. Prior to this, a pilot study was conducted using 15 specimens in each of 3 treatment groups (3 specimens from each of the 5 tested resin composites). Means and standard deviations were calculated for the dependent variables and were used to calculate the sample size using web-based OpenEpi software (www.openepi.com). Results showed that the sample was adequate for a confidence interval of 95% and 80% power. Therefore, additional specimens were not necessary.

**Table 1:**
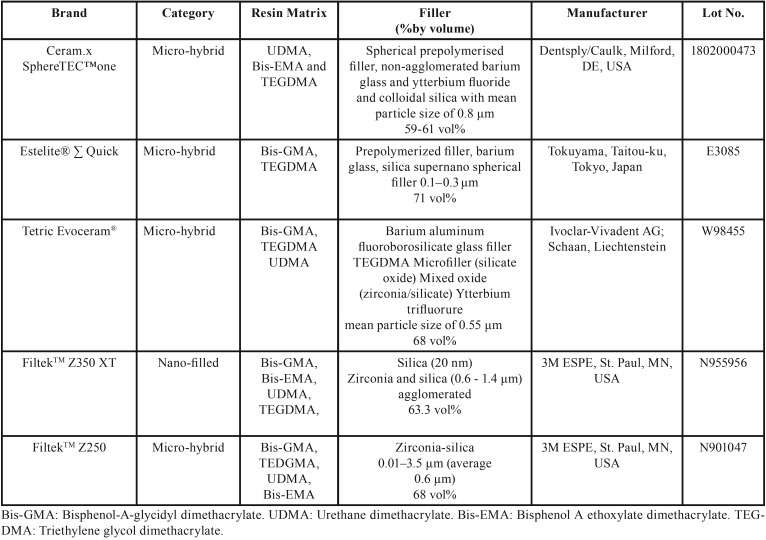
Resin composites tested in the study.

The specimens were prepared by condensing the material into an extra firm silicone mold fabricated from (Express™ VPS Bite Registration Material Putty; 3M ESPE, St Paul, MN, USA) with size of 6 mm in diameter and 2 mm in thickness closed from the bottom with a polyester strip and glass slide. The mold was filled with the composite resin and a second polyester strip was placed on the top of the filled mold. A glass slide was placed against the upper polyester strip and pressed for 30 seconds by placing standardized 500 g weight to extrude the excess composite resin and to form a flat surface. The material was then light-cured from the top according to the manufacturers’ recommendations (for 20 seconds) by placing the head polymerizing light over an in flush contact with the surface of the glass slide and concentrically positioned over the packed specimen in the silicon mold. a light-emitting diode (LED) visible light-polymerizing unit (SmartLite Max LED Curing Light; Dentsply Sirona, Milford, DE, USA) was used in continuous mode (1200 mw/cm2 light intensity) for curing of the specimens. The Built-in radiometer in the light cure unit was used to verify the power output of the curing light source before photo-polymerization of each specimen.

Once polymerized, the specimens were dislodged from the mold and then the top surfaces and sides of all specimens were then polished all in the same direction parallel to the surface and in standardized manner to remove the resin-rich surface layer using fine (24 μm) and superfine (8 μm) aluminum oxide polishing disks (Sof-lex, 3M ESPE, St Paul, MN, USA) with a slow-speed hand piece rotating at 35,000 rpm under constant vertical load of 200g. Next, the specimens were cleaned in distilled water ultrasonic cleaner bath for 5 minutes and stored in distilled water for 7 days in an incubator at 37°C.

-Specimen treatment measurements

**Figure 2:**
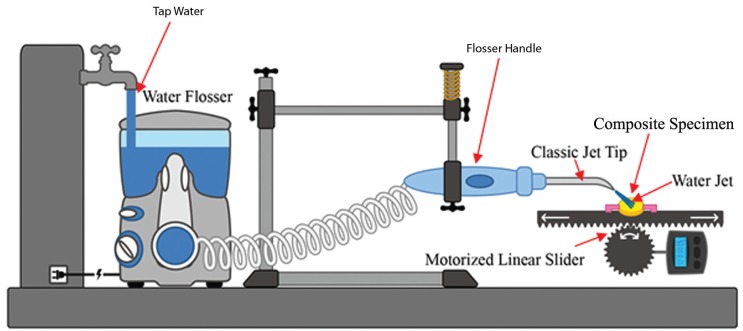
Schematic diagram of test apparatus.

For each composite, the specimens were randomly allocated into three groups: storage in distilled water (control), water flossing at 50 Psi water pressure and water flossing at 100 Psi water pressure. The water flosser was an ADA-accepted Waterpik® Aquarius® (Water Pik, Inc. Fort Collins, CO, USA). The water flossing process was standardized using a customized precision slider assembly used in a previous study (22). The specimen was first fixed in the mini vise of the slider with the treatment surface facing upward. The slider was then programmed to move 3 mm continuously in each direction with the flosser handle fixed to the vertical arm of the assembly with the tip (Waterpik® Classic Jet Tip) opening was at distance of 2mm from the specimen’s surface and the angle between the treatment surface and the water jet at 45° (Fig. 2). The slider motor was activated so the specimen moved 3 mm in each direction under the jet tip. Next, the flosser was turned on to blast the specimens with water jet while the flosser reservoir was continuously filled with drinking tap water. The water flosser was used in flosser mode at two different pressure settings: the full/highest pressure setting (approximately 100 Psi) and half of the full pressure setting (50 Psi) according to treatment group. A total of 45 specimens in the three treatment groups were evaluated for color changes and surface roughness at two different intervals: baseline and after the end of 30 minutes of treatment, which is equivalent to 5 years of simulated water flossing for 1 minute once a day. Color measurements were obtained with a spectrophotometer (Vita Easyshade, Vita Zahnfabrik, Bad Säckingen, Germany) and expressed as Commission Internationale d’Eclairage (CIE) L*a*b* coordinates. The measurements were performed by a single operator according to the manufacturer’s instructions. First the instrument was calibrated before use on each specimens using calibration block attached to base unit then the measurements were performed on each specimen against a neutral gray background (X-rite ColorChecker Passport, X-rite Inc.). Surface roughness was measured using a bench-top optical surface-profiling system (Optical Profilometry Contour GT-I 3D Optical Microscope; Bruker Daltonics Inc., Billerica, MA, USA). The L*a*b* color coordinates and surface arithmetical mean height (Sa) of each specimen at the two measurement points were tabulated.

-Statistical analysis

The total color difference (∆E) between the specimen’s baseline measurement and the after-treatment measurement was calculated using the CIE76 color difference formula. To determine the difference in surface roughness before and after treatment, the arithmetical mean height of the surface at baseline (Sab) was subtracted from the surface arithmetical mean height after the treatment (Sat); ∆Sa was expressed in micrometers (μm). After verifying the normality of the results with the Shapiro-Wilk test and homogeneity of variances using Levene’s test, a two-way analysis of variance (ANOVA) was conducted to examine the effects of composite type and treatment on color change and surface roughness of the composite resins. A Bonferroni correction and Tukey’s post-hoc tests were used to determine statistically significant differences among the groups. The statistical analysis was performed using SPSS 20.0 software (Statistical Package for the Social Sciences, SPSS Inc., Chicago, IL, USA).

## Results

Two-way ANOVA was used to examine the effects of composite type and water-jet flossing on changes in color and surface roughness score. Residual analysis was performed to test for the assumptions of the two-way ANOVA. Outliers were assessed by inspecting a boxplot, normality was assessed using Shapiro-Wilk’s normality test for each cell of the design and homogeneity of variances was assessed using Levene’s test. There were no outliers, the residuals were normally distributed (*p* > .05) and there was homogeneity of variances (*p* = 0.10 and *p* = 0.08, respectively)

There was no statistically significant effect of composite type and water-jet pressure on the change in color (∆E) (*p* > 0.05), and all color changes were within the clinically accepTable value of ∆E ≤ 3.3 ( Table 2).

**Table 2:**
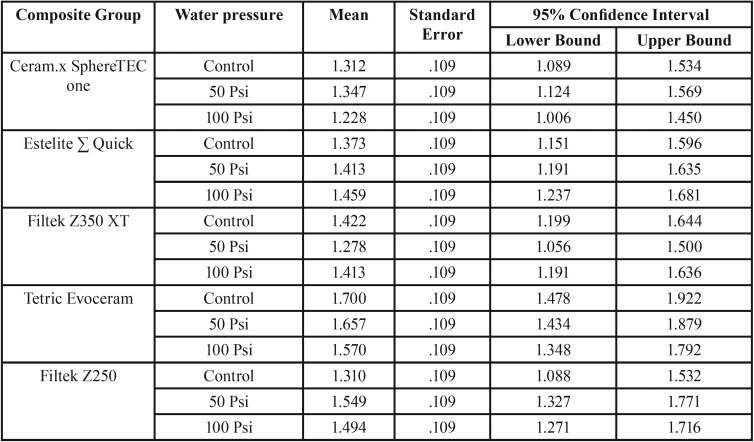
Mean change in color ΔE values and 95% Confidence Interval for the five tested composites in three treatment groups.

There was no statistically significant main effect of composite type on the change in surface roughness score: F (4,30) = 2.390, *p* = 0.073, partial η2 = 0.242; the main effect of water-jet flossing was statistically significant: F (2,30) = 25.981, *p* < 0.001, partial η2 = 0.634. There was also a statistically significant interaction between composite and water-jet flossing in terms of a change in surface roughness score: F (8,30) = 2.454, *p* = 0.036, partial η2 = 0.396. The mean change in tested resin composites surface roughness values (∆Sa) are presented in  Table 3.

**Table 3:**
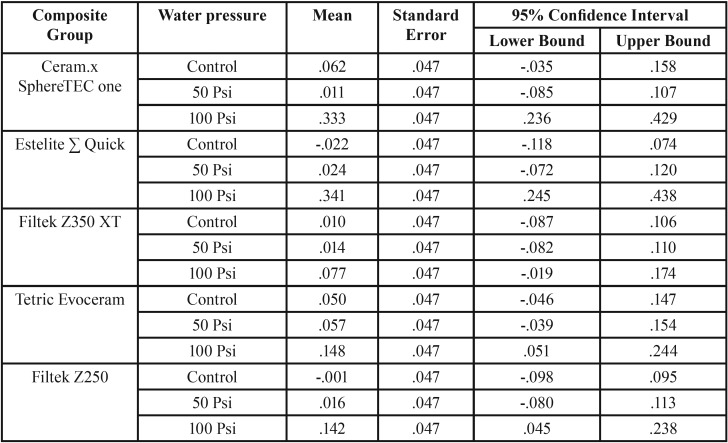
Mean change in surface roughness values (∆Sa) and 95% Confidence Interval for the five tested composites in three treatment groups.

Pairwise comparisons were run for each simple main effect with reported 95% confidence intervals and p-values. Bonferroni-adjusted statistical significance within each simple main effect revealed that the100 Psi water-jet pressure group was associated with a higher roughness mean score than both the control group and the 50 Psi group (.188 μm, 95% CI [.112, .264], *p* < 0.001; .183 μm, 95% CI [.108, .259], respectively), which was a statistically significant difference (*p* < 0.001).

Due to a significant interaction between composite type and water-jet flossing in terms of a change in surface roughness score, an analysis of the simple main effects of water-jet pressure was performed. The statistical significance received a Bonferroni adjustment and was accepted at the *p* < 0.025 level. There was a statistically significant difference in mean change in surface roughness scores between the 100 Psi water pressure group in terms of different composite types: F (4,30) = 6.575, *p* = 0.001, partial η2 = 0.467. Pairwise analysis showed that both Ceram.x and Estelite Sigma specimens in 100 Psi treatment group exhibited a significantly higher increase in surface roughness when compared with Z350 specimens ( Table 4). The simple main effects for composite type reveled that there was a statistically significant difference in the change in surface roughness score between different water-jet flossing groups for only Ceram.x and Estelite Sigma: F (2,30) = 13.467, *p* < 0.001, partial η2 = 0.473; F (2,30) = 17.623, *p* < 0.001, partial η2 = 0.540, respectively. However, for the other composite types this difference was not statistically significant.

**Table 4:**
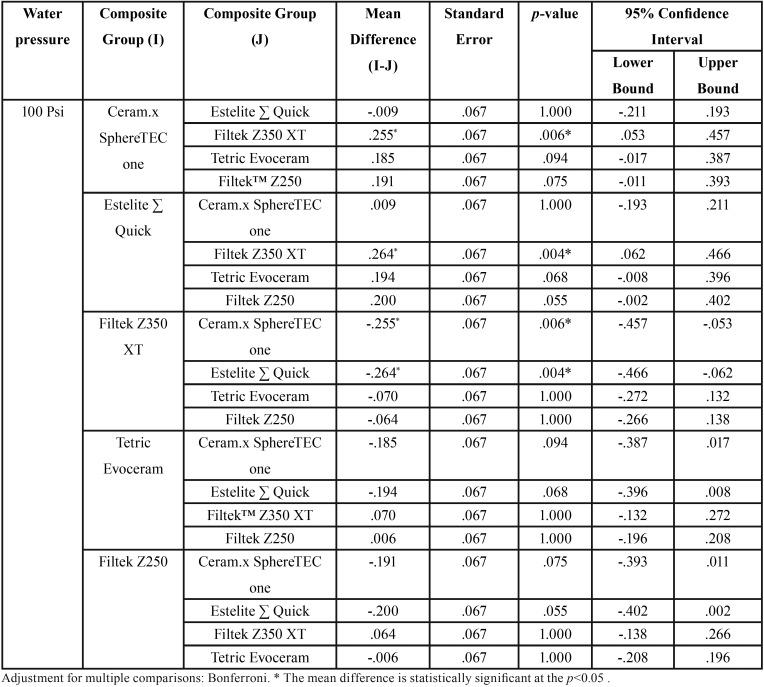
Pairwise comparison of mean change in surface roughness values (∆Sa) between the five tested composites in 100 Psi treatment group.

## Discussion

This study examined the effects of water jet flossing on several contemporary resin-based composites using the results of color stability and changes in surface roughness.

To standardize the specimens, all composites were used in A2 shade and the final polishing step was done with discs with abrasive particles 8 μm in size; this size is recommended to attain a clinically accepTable surface roughness and gloss (23). This procedure yielded a standard baseline reference point to start with and remove the weak composite surface layer rich in resin matrix that resulted from composite polymerization against the polyester strip, which may, if present, have affected the results (24). The treatments were conducted in a standardized manner using a slider assembly for a time equivalent of 5 years; longevity and survival studies have shown that dental resin composites available within the past 10 years have an average replacement time of 5.7 years (25).

To obtain a quantitative description of color consistent with previous studies, the L*, a* and b* color attributes in CIELAB color space were used to express composite color. For surface roughness, we used the arithmetical mean height of the surface (Sa), which is equivalent to the arithmetical mean height of the line (Ra) used in most previous surface roughness studies (26). Because composites’ color and surface quality are a reflection of their composition and filler characteristics, which are brand dependent, there was variation in the initial measurements between different types of composites (27,28). For the color coordinates, the highest L (lightness) and b (blue-yellow axis) coordinates were noted in Ceram.x SphereTEC™ (L=85.6 and b=20.4); the lowest values were noted in Estelite Sigma Quick (L=78.6; b=12.4). For a coordinate (green-red axis), the lowest was noted for Ceram.x SphereTEC™ (a=-2.8); the highest value was noted for Tetric Evoceram (a=1.2). This finding emphasizes that when a clinician selects a composite shade there is no standardization in composites’ colors between different manufacturers; a manufacturer’s own shade standard should be used.

There was also variation in the initial surface roughness of different composites despite the standardized polishing technique. These differences may be due to intrinsic composite composition factors such as the filler (type, shape, size, hardness and distribution of the particles), the type of resin matrix, the degree of polymerization and the bond efficiency at the filler/matrix interface. The values of initial surface roughness obtained in this study, agree with the values obtained in other studies in which the roughness values ranged from 0.3–1.2 μm (29,30). The smoothest composite was the nano-filled composite (Sab = 0.426 μm); the roughest one was the micro-hybrid (Sab = 1.049 μm). This finding is consistent with previous studies that found that smaller filler sizes resulted in lower surface roughness values after abrasion polishing (26). Therefore, the nano-filled composite exhibited lower roughness values than the Submicron and micro-hybrid composites. The roughness values reported in this study was higher than in other studies, which is common given the variations in these types of studies. This finding may be due to technique-related problems, which are inherently prone to mistakes linked to specimens’ fabrication techniques, polishing techniques, measurement techniques or the devices used to take the measurements.

The first null hypothesis was accepted: water flossing did not affect the color stability of the composites used, irrespective of the type of composite or the water pressure. Furthermore, the color change in all specimens was imperceptible (∆E ≤ 2). This finding is consistent with the results of previous studies that water storage and water sorption alone did not alter the composites’ colors to a considerable extent (31,32). Additionally, theoretically, the surface texture normally affects the color coordinates because it affects the amount, direction and quality of reflected light. However, in this study the variation in the surface texture of all specimens was less than the wavelength of visible light (i.e., approximately 0.5 μm). Therefore, slight roughness changes in surface had no effect on the reading of the spectrophotometer (33).

**Figure 3:**
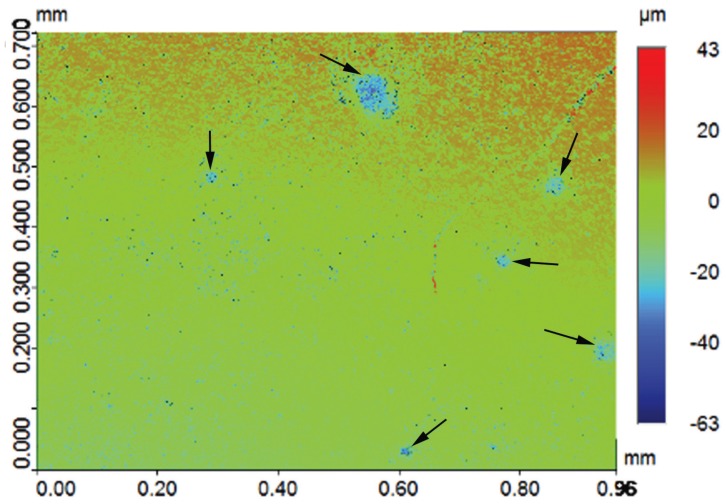
Optical surface profilometer two-dimensional (2D) surface topography image of the 100 Psi group reveal deep valleys (black arrows) resulting from the repeated water-jet impact.

In terms of our second null hypothesis, the results were not clear cut: the mean changes in surface roughness for the 50 Psi group were not significantly different from those of the control group for all composite types. Therefore, water flossing at 50 Psi did not affect the surface roughness of any of the types of composites. On the other hand, the mean change in surface roughness in the 100 Psi group was statically higher than that of both the control group and the 50 Psi group ( Table 3). The effect of 100 Psi water flossing was not uniform on all of the composite groups; the Estelite Sigma Quick and Ceram.x composites were the most poorly affected by the high-pressure flossing. These composites exhibited a mean increase in surface roughness of 0.341 and 0.333 μm, respectively. This increase was slightly more than the 0.2 μm threshold suggested in the literature for a substantial increase in bacteria colonization proposed by Bollen *et al.* (34). The composite group least affected by the high-pressure flossing was the nano-filled (Z350) composite, which exhibited a mean increase in surface roughness of 0.077 μm. The Z250 composite exhibited an increase of 0.142 μm, and the Tetric Evoceram composite exhibited an increase of 0.148 μm. The only significant differences were between the Estelite Sigma Quick and Ceram.x SphereTEC™ from one side and Z350 from the other side. This result can be explained by the fact that Estelite Sigma Quick and Ceram.x SphereTEC™ both have comparatively large size spherical shape fillers (0.2–3.5 μm), which can be pluck from it housing resin matrix under the shear force of the water jet impacting; repeated stresses lead to fatigue failure of the bond at filler/matrix interface. This explanation is supported by the deep valleys noted in some three-dimensional microscope images of the Estelite Sigma Quick and Ceram.x SphereTEC™ in the 100 Psi group (Fig. 3). On the other hand, composites with irregularly shaped fillers buried deep in resin matrix undercuts were less likely to be dislodged under the impact of the water jet. For the nano-size filled composite, the high well distributed filler loading and very small-sized filler particles (20 nm) resulted in less distance between the filler particles that the impact stress. As a result, the impact stress was not concentrated on the filler alone and was distributed and absorbed by both the housing resin matrix and multiple fillers. This situation led to increased stability of the fillers and less stress on the bond at the filler/matrix interface (35,36).

There are limitations to this *in vitro* study. Every effort was made to simulate real *in vivo* flossing conditions—such as angle of water-jet impact, pressure and distance from tip—but we were unable to fully simulate the dynamic oral environment and its fluctuations in pH, masticatory forces, and the presence of bacteria and saliva. The uniform flat surface of the specimen may have produced different outcomes after flossing than the curved contours of the restoration, which would have changed the force vector of the impacting water-jet. No standardized *in vitro* protocol has been established to resemble the complexity of the oral environment. Therefore, future studies that include more parameters are necessary to simulate *in vivo* flossing. The addition of aged composites will also be beneficial. Furthermore, clinical observations of restorations in the mouth will shed light on the impact of water-flossing and changes in color and surface roughness of composite resins. Despite these limitations, this study yielded new knowledge: water-jet flossing on composite resins does not severely compromise the roughness or color stability. These findings will reassure dentists about prescribing these devices and the use of this prophylactic agent in patients with smooth surface composite restorations.

## Conclusions

Within the limitations of this study, the use of water-jet flossing is safe for composite restorations. However, patients should be advised not to continuously use the highest-pressure setting when they have restored teeth with resin composite restorations.
